# Bilateral serous surface papillary borderline ovarian tumor with non-invasive implantation: a case report

**DOI:** 10.3389/fonc.2026.1705073

**Published:** 2026-01-30

**Authors:** Zhengping Wang, Sumei Gao, Shuli Wei, Yina Jiang, Mengling Li

**Affiliations:** 1 Department of Ultrasound, Weifang People's Hospital, Weifang, Shandong, China; 2 Department of Pathology, Weifang People's Hospital, Weifang, Shandong, China

**Keywords:** borderline ovarian tumors, external tumor of ovary, non-invasive implantation, ovarian neoplasms, serous surface papillary borderline tumors, ultrasonography

## Abstract

**Introduction:**

Serous surface papillary borderline ovarian tumors (SSPBOTs), a clinically distinct variant of serous borderline ovarian tumors (SBOTs), are frequently misdiagnosed as malignant tumors during preoperative diagnostic assessments.

**Presentation of case:**

This case report describes a 22-year-old female patient who presented with abdominal pain. Gynecological examination revealed a painless 11-cm diameter mass on the right posterior side of the uterine body. Ultrasonography demonstrated a solid mass in the right ovary (O-RADS 5, highly indicative of malignant germ cell tumor). It also revealed a unilocular cystic mass in the left ovary (O-RADS 2, with benign characteristics). During the operation, a cauliflower-like mass was observed on one side of the surface of the right ovary. On the left ovary, a cystic mass was found, with multiple small cauliflower-like masses scattered on its surface. Additionally, pelvic cavity examination revealed millet-sized lesions and fibrinous exudate. The pathological diagnosis confirmed the presence of bilateral serous borderline tumors with multiple non-invasive implants. Treatment involved the excision of bilateral ovarian masses and the resection of secondary lesions. No evidence of recurrence was observed during the one-year follow-up.

**Discussion:**

SSPBOTs are a distinct subtype of serous borderline ovarian tumors that predominantly affecting women of reproductive age and often involve bilateral ovaries. Ultrasound findings mostly show solid hypoechoic masses growing on the surface of the ovary. These masses surround normal ovarian tissue and exhibit a visible “microcystic sign” within. Punctate strong echoes or coarse calcified spots may also be observed. Color Doppler ultrasound demonstrates abundant intratumoral vascularity with a ‘firework-like’ perfusion pattern. Insufficient understanding of this disease can easily lead to misdiagnosis as a germ cell tumor. Although CA-125 levels may be elevated, the marker lacks diagnostic specificity. Given the tumor’s indolent growth pattern and low recurrence rate, fertility-sparing surgery is feasible for patients desiring reproductive preservation.

**Conclusion:**

This report systematically summarizes the ultrasonographic characteristics of SSPBOTs, aiming to enhance diagnostic accuracy and provide evidence-based support for fertility preservation strategies in young patients.

## 1 Introduction

Serous borderline ovarian tumors (SBOTs) are the most common subtype of borderline ovarian neoplasms, characterized by morphological features intermediate between benign serous tumors and low-grade serous carcinomas (1). Serous surface papillary borderline ovarian tumors (SSPBOTs) constitute a distinct histological subtype of SBOTs, predominantly affecting reproductive-aged women. These tumors are typically associated with bilateral ovarian surface involvement and the presence of invasive or non-invasive extraovarian implants, which often leads to preoperative misdiagnosis as malignant neoplasms. Here, we report a case of SSPBOT with non-invasive implantation, aiming to enhance clinical awareness of this entity and reduce the incidence of misdiagnosis and missed diagnosis.

## 2 Presentation of case

**Figure 1:**
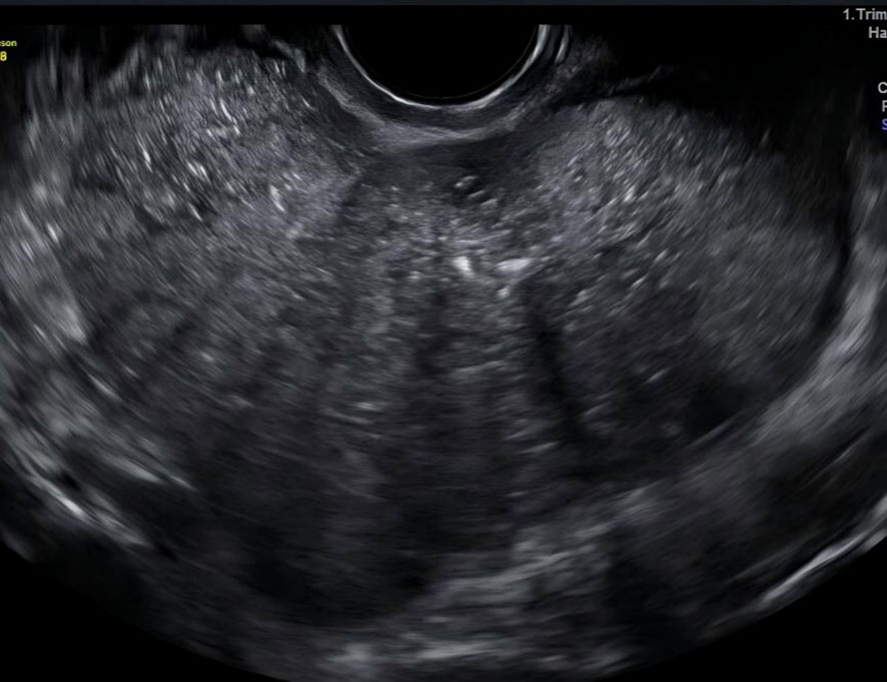
Multiple coarse hyperechoic spots and dense small anechoic areas within the right ovarian mass.

**Figure 2:**
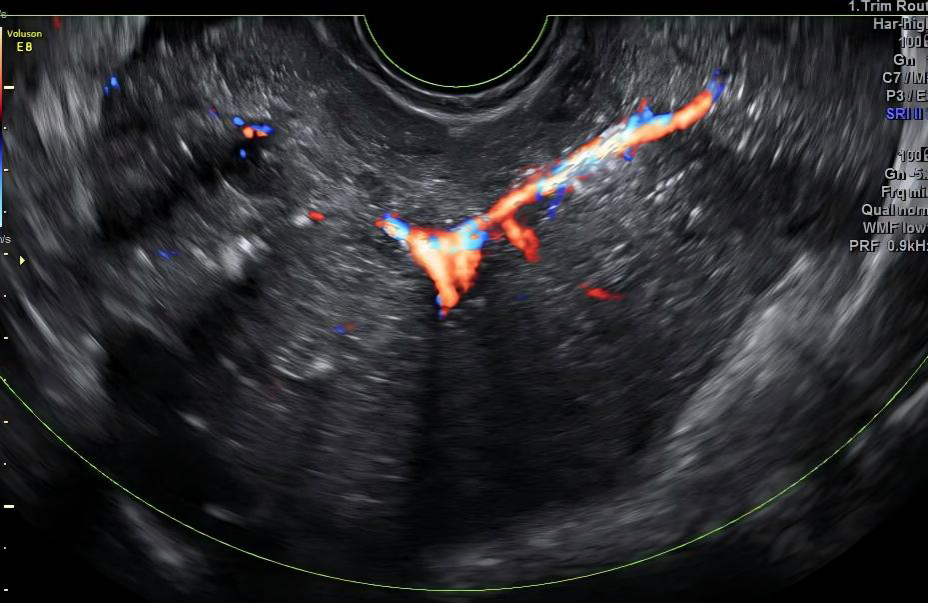
Branched strip-like blood flow signals within the right ovarian mass.

A 22-year-old woman presented to our hospital with abdominal pain for evaluation. Gynecological examination revealed a painless 11-cm diameter mass on the right posterior side of the uterine body. Transvaginal ultrasound demonstrated: a normal-sized uterus with regular morphology; a 10.8 cm × 7.3 cm hypoechoic mass in the pelvic cavity, exhibiting well-defined borders, irregular shape, heterogeneous echogenicity, multiple coarse hyperechoic spots, and densely distributed small anechoic areas (**Figure 1**). Color Doppler flow imaging (CDFI) showed abundant branching vascular signals within the solid component (**Figure 2**), with a pulsatility index (PI) of 0.42;The left ovary contained a 4.3 cm × 4.0 cm anechoic cystic lesion with fine septations and poor sound transmission. Free fluid in the rectouterine pouch, measuring 2.8 cm in depth, showed poor sound transmission. Ultrasound impression: 1. Solid pelvic mass (O-RADS 5), likely originating from the right ovary, suggestive of a germ cell tumor; 2. Left ovarian cystic lesion (O-RADS 2); 3. Pelvic effusion. Laboratory findings: Serum CA125: 602 U/mL (normal range: 0–47 U/mL). The patient reported no menstrual changes, abnormal vaginal bleeding or discharge, dizziness or fatigue, or significant weight changes since the onset of the disease. The patient denied a history of major illnesses or surgeries. She was unmarried, had a sexual history, had no prior pregnancies, and had a regular menstrual cycle.

**Figure 3:**
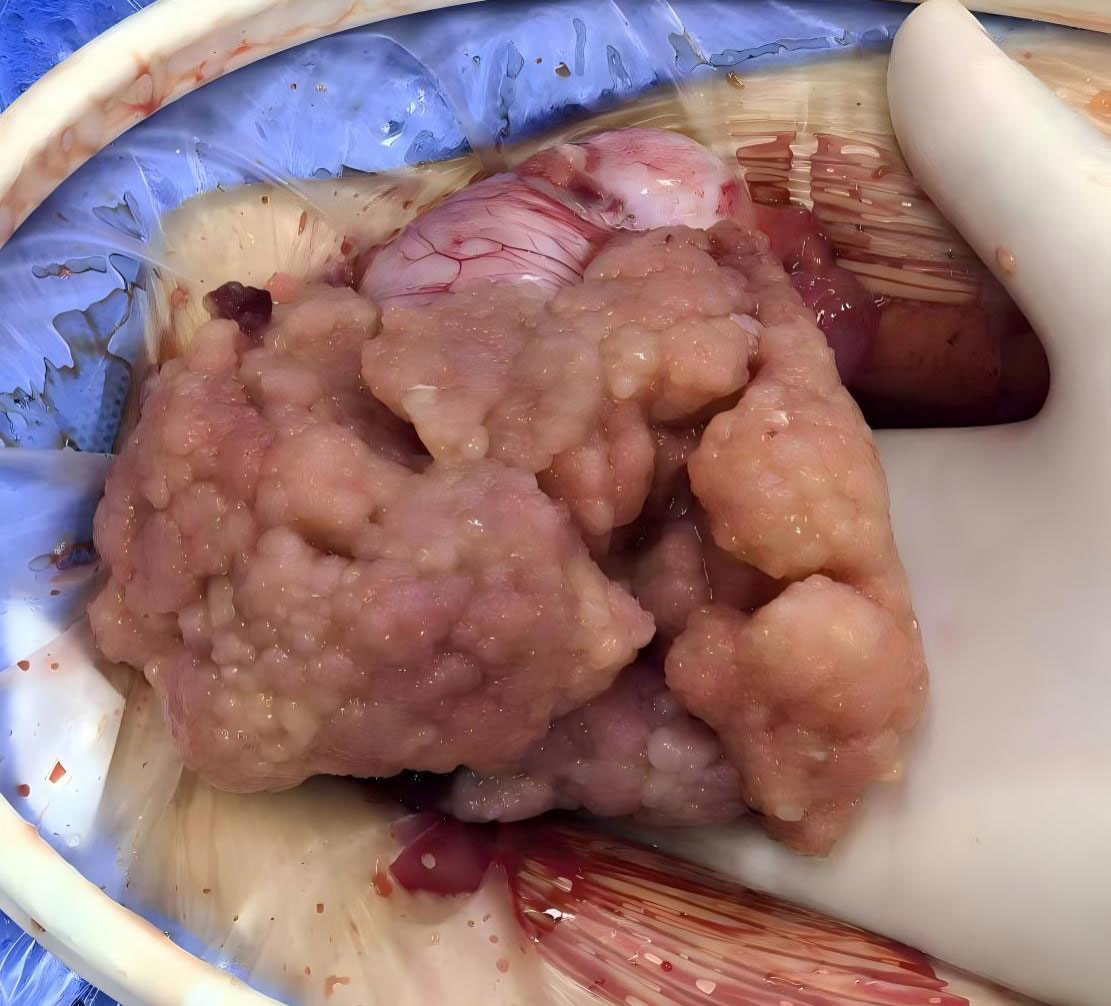
The cauliflower-like mass of right ovay.

**Figure 4:**
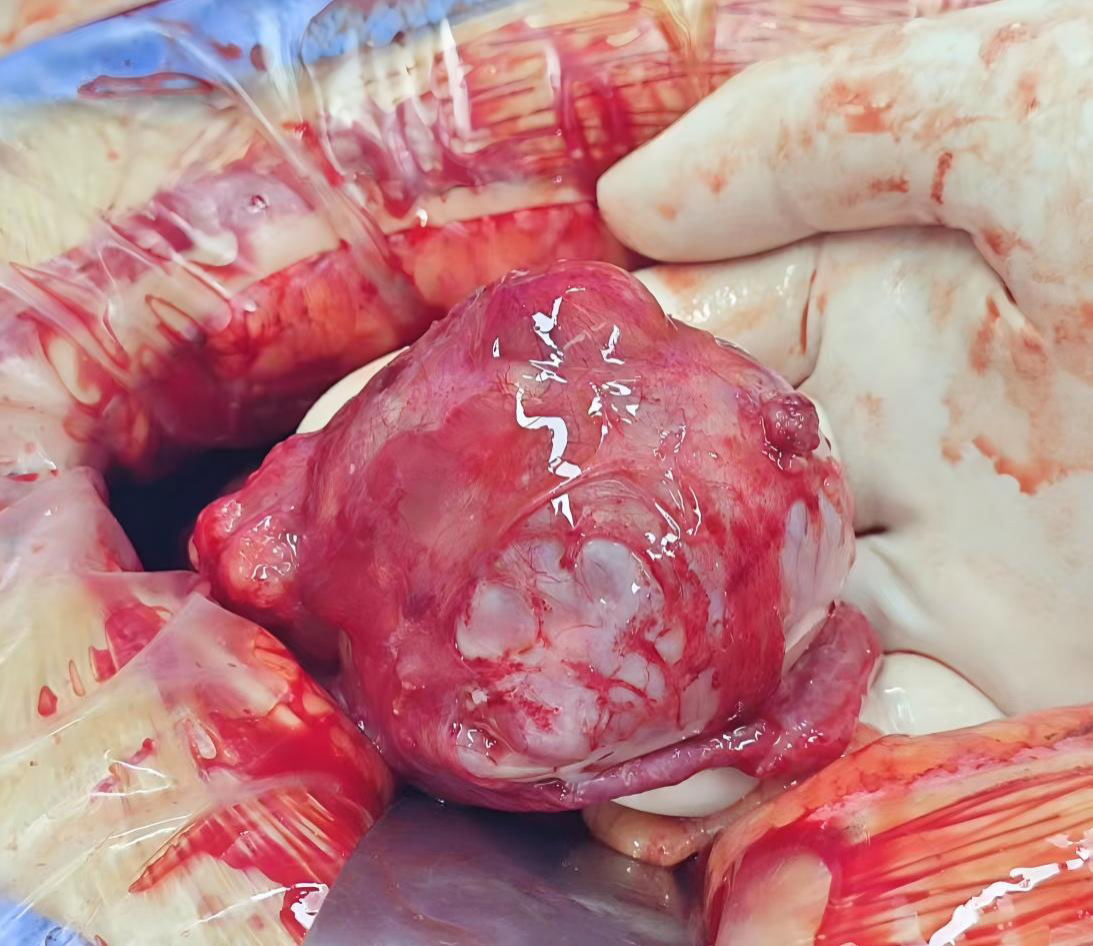
The cauliflower-like masses on the surface of the left ovary.

**Figure 5:**
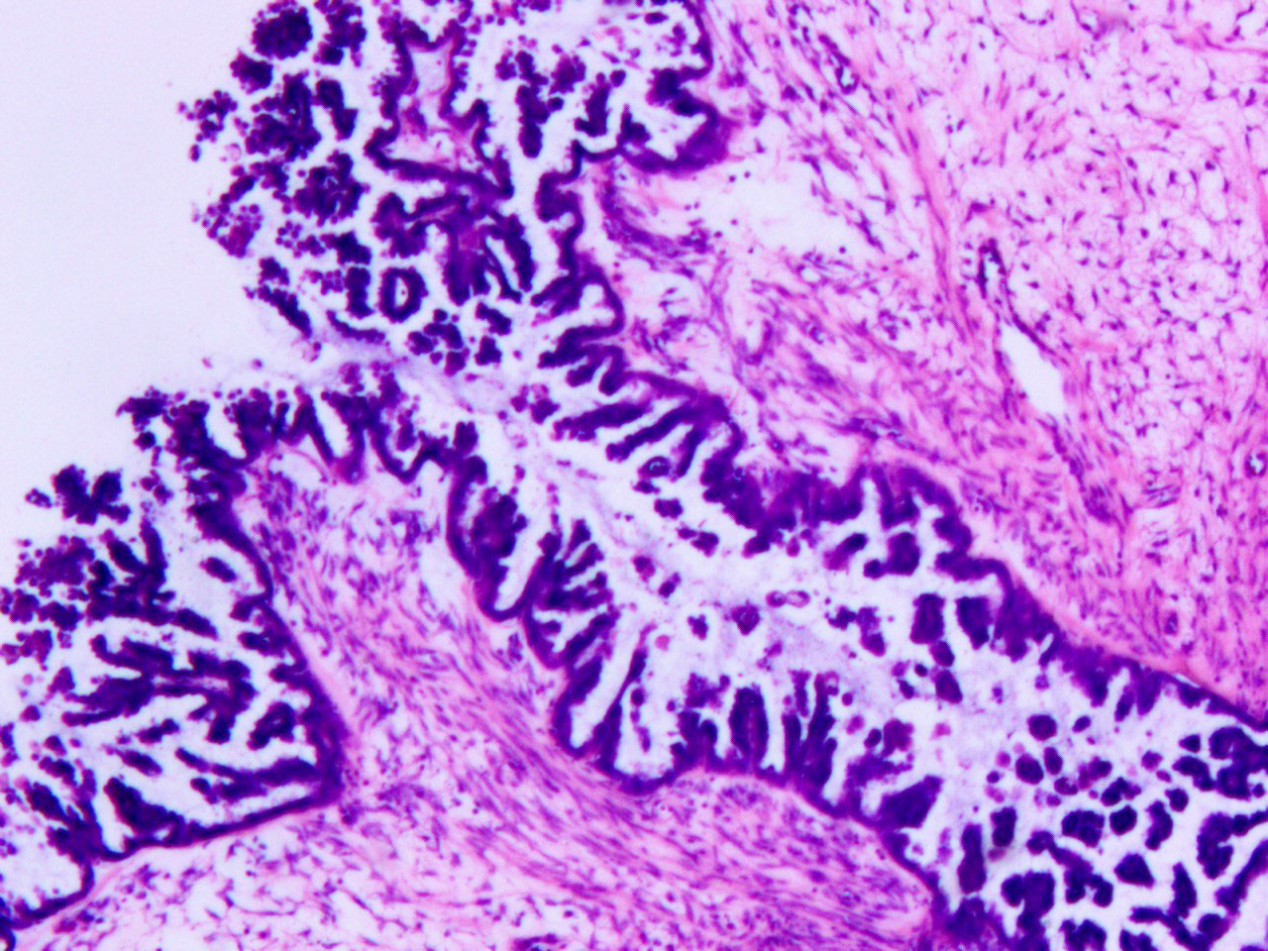
The pathological image of right ovarian mass (4x).

Following confirmation of the surgical indications in the preoperative evaluation, laparoscopic exploration was performed as the first step. Intraoperative Findings: Approximately 150 mL of yellowish ascitic fluid was present in the abdominopelvic cavity; The uterus was normal in size but was covered by fibrinous exudates and scattered small cauliflower-like nodules, the largest measuring 0.5 cm in diameter; The right ovary was enlarged to 15 cm and showed cystic-solid components, multiloculated structure, and an irregular surface with a cauliflower-like growth at one pole (**Figure 3**); The left ovary measured 5 cm in diameter with cystic-solid morphology and multiple small surface nodules (**Figure 4**); Extensive fibrinous exudates were noted on the peritoneal surfaces, the rectal and colonic mesentery, and the bladder. ‘Harp-string’ adhesions bridged the liver and peritoneum. The omentum contained miliary nodules, and the paracolic gutters showed filmy fibrinous deposits. In view of this situation, the surgical team promptly informed the patient’s family of the intraoperative exploration results. They also learned that the patient strongly wished to preserve fertility and explicitly requested an open surgery. Therefore, a midline longitudinal incision slightly to the left of the lower abdomen was subsequently made to further explore the ovarian space-occupying lesions and the specific conditions of the pelvic and abdominal organs. Considering the family’s desire to preserve fertility, the surgical team first performed bilateral ovarian tumor enucleation and resection of part of the fibrous tissue in the pelvic cavity, and sent the resected tissue for frozen section pathology examination. The pathological results showed that the right and left ovarian tumors were consistent with borderline serous cystadenoma; borderline serous cystadenoma components were found in the pelvic fibrous tissue (**Figure 5**). Due to the patient and family’s strong desire for fertility preservation, the following procedures were performed: bilateral ovarian mass excision, resection of uterine surface tumors, excision of bilateral pelvic wall peritoneal tumors, resection of anterior rectal wall tumors, and greater omentectomy. Postoperative follow-up has lasted for approximately 1 year with no recurrence observed.

## 3 Discussion

The 2014 WHO Classification of Tumours of the Female Reproductive System histologically defines borderline ovarian tumors (BOTs) as “atypical proliferation of ovarian epithelial cells without stromal invasion,” specifying that the borderline component should constitute more than 10% of the tumor mass. BOTs account for 15–20% of ovarian epithelial neoplasms, encompassing serous, mucinous, seromucinous, endometrioid, clear cell, and Brenner subtypes. SBOTs represent the most common BOT subtype, predominantly affecting reproductive-aged women (1). Serous epithelial ovarian neoplasms are classified by proliferative activity into cystic, surface proliferative, and stromal invasive types (2). SSPBOTs, a distinct subtype of SBOT, are characterized by ovarian surface growth, younger age at presentation, and nonspecific clinical symptoms. Patients often present with large abdominal solid masses, which make preoperative diagnosis challenging and primarily dependent on intraoperative findings and histopathological examination. Given their low malignant potential and predilection for young women, early detection and management are critical for implementing fertility preservation strategies.

Grayscale, color and power Doppler ultrasonography are the initial imaging modalities for characterizing adnexal masses and evaluating their risk of malignancy (3). Endovaginal scanning, using high-frequency probes, enables superior visualization of tumor surface micropapillary structures and internal architecture. Moreover, its real-time blood flow imaging capability provides comprehensive data on intratumoral vascular signals and resistance index (RI). As proposed by Ludovisi et al (4),ultrasound is the only modality aiding differentiation between SSPBOT solid tumors and other malignant lesions, by identifying the presence of normal ovarian tissue partially or completely encased by the solid mass. Ultrasound imaging of SSPBOTs typically demonstrates solid hypoechoic lesions arising from the ovarian surface, with partial or complete encasement of normal ovarian parenchyma. Key sonographic features include “microcystic signs”, punctate or coarse calcifications, and “firework-like” vascularity on color Doppler imaging, reflecting abundant intratumoral branching blood flow. Recent studies have proposed the “microcystic sign” as a novel ultrasonographic marker for diagnosing BOTs (5). This characteristic feature is defined as densely clustered, thin-walled microcystic structures measuring 1–3 mm in diameter within the tumor tissue. The presence of this sign, particularly when combined with other characteristic findings such as surface papillary projections and moderate vascularity (RI 0.4-0.6), may significantly improve the preoperative diagnostic accuracy for BOTs. MRI demonstrates branched low-signal structures within the solid tumor, exhibiting an “anemone-like” appearance (6). In this case, the right ovarian tumor presented as a primarily exophytic solid mass, with multiple calcifications, microcystic signs, and branching vascular bundles, exhibiting all the typical ultrasound features of SSPBOTs; however, we still misdiagnosed it, primarily due to insufficient understanding of this condition and lack of careful observation, which caused us to fail to notice the presence of normal ovarian tissue. The left ovarian borderline serous tumor presented as a unilocular cyst with poor acoustic transmission. Owing to the small volume of surface nodules, ultrasound imaging failed to visualize them, thereby leading to the misclassification of the cyst as an O-RADS category 2 lesion. The difference between the huge solid hypoechoic tumor of the right ovary and the unilocular cystic tumor of the left ovary in this case highlights the complexity of the ultrasound manifestations of the disease. These findings underscore that enhancing awareness of SSPBOTs and implementing meticulous observation of all ovarian neoplasms during the diagnostic workup are critical for improving diagnostic accuracy. Furthermore, this case demonstrates the inherent limitations of ultrasound in resolving submillimeter pathological features, underscoring the need for complementary MRI in equivocal cases.

CA-125, CA-199, and CEA are the most widely utilized tumor markers in gynecological malignancies. Previous studies have established that CA-125 is predominantly overexpressed in patients with serous ovarian carcinoma (7). Consistent with these findings, the current case demonstrated elevated CA-125 levels, further supporting its association with this tumor subtype. However, CA-125 secretion is influenced by multiple non-neoplastic conditions, including endometriosis and pelvic inflammatory disease, which may also contribute to moderate-to-marked CA-125 elevation (8). Therefore, differential diagnosis is essential to distinguish malignancy from benign gynecological pathologies when interpreting CA-125 levels.

The optimal management of SSPBOTS remains controversial, with treatment strategies primarily guided by tumor characteristics, disease stage, and the patient’s fertility-preservation goals. Given the indolent growth pattern and favorable prognosis of borderline tumors—where recurrences are typically non-invasive and remain borderline in nature—fertility-sparing surgery (FSS) is a viable option for patients desiring future childbearing. The recurrence rate after fertility‐preserving surgeries for BOT (which varies between 5 and 34%) is higher than the recurrence rate after more radical surgeries (reported to be between 3.2 and 7%) (9). However, with close surveillance and extended follow‐up, most recurrent BOT cases after ovarian preserving approaches are safely managed surgically with good oncologic outcomes (10). In the present case, the patient and her family strongly prioritized fertility preservation. Since the peritoneal implants exhibited non-invasive histology, a conservative surgical approach was pursued, involving bilateral ovarian cystectomy with excision of secondary lesions. No evidence of recurrence was detected during the 12-month follow-up period, further supporting the safety of FSS in selected patients.

While this case provides valuable insights for relevant clinical practice, it is not without limitations. First, the small sample size of included cases precludes the analysis of differences in the imaging features of SSPBOT across populations of different ethnicities and regions. Second, the technical proficiency and clinical diagnostic experience of ultrasound practitioners may also impact the accuracy of the final diagnosis. Nevertheless, by summarizing and reflecting on the clinical characteristics of this case as well as its diagnostic and management strategies, this study aims to offer directions for future research on related topics, thereby contributing modestly to improving the early diagnosis rate of SSPBOT and enhancing patient prognosis.

## 4 Conclusion

SSPBOTs are a rare entity, yet their unique clinical behavior underscores the critical importance of early diagnosis and tailored treatment strategies, particularly for young patients. Transvaginal ultrasound (TVUS) serves as the primary non-invasive diagnostic modality for ovarian tumor evaluation, offering real-time assessment of tumor morphology and internal vascularity through Doppler imaging. These sonographic features provide valuable insights into the tumor’s biological behavior, aiding in differential diagnosis and risk stratification. This report aims to raise awareness of this condition, reduce misdiagnoses, and offer the best treatment options for patients.

## Data Availability

The original contributions presented in the study are included in the article/supplementary material. Further inquiries can be directed to the corresponding author.
